# A Novel Carbonyl Reductase with Anti-Prelog Stereospecificity from *Acetobacter* sp. CCTCC M209061: Purification and Characterization

**DOI:** 10.1371/journal.pone.0094543

**Published:** 2014-04-16

**Authors:** Xiao-Hong Chen, Ping Wei, Xiao-Ting Wang, Min-Hua Zong, Wen-Yong Lou

**Affiliations:** 1 Lab of Applied Biocatalysis, College of Light Industry and Food Sciences, South China University of Technology, Guangzhou, Guangdong, China; 2 State Key Laboratory of Pulp and Paper Engineering, South China University of Technology, Guangzhou, Guangdong, China; Università degli Studi di Milano, Italy

## Abstract

A novel carbonyl reductase (*Ac*CR) catalyzing the asymmetric reduction of ketones to enantiopure alcohols with anti-Prelog stereoselectivity was found in *Acetobacter* sp. CCTCC M209061 and enriched 27.5-fold with an overall yield of 0.4% by purification. The enzyme showed a homotetrameric structure with an apparent molecular mass of 104 kDa and each subunit of 27 kDa. The gene sequence of *Ac*CR was cloned and sequenced, and a 762 bp gene fragment was obtained. Either NAD(H) or NADP(H) can be used as coenzyme. For the reduction of 4′-chloroacetophenone, the K_m_ value for NADH was around 25-fold greater than that for NADPH (0.66 mM *vs* 0.026 mM), showing that *Ac*CR preferred NADPH over NADH. However, when NADH was used as cofactor, the response of *Ac*CR activity to increasing concentration of 4′-chloroacetophenone was clearly sigmoidal with a Hill coefficient of 3.1, suggesting that the enzyme might possess four substrate-binding sites cooperating with each other The V_max_ value for NADH-linked reduction was higher than that for NADPH-linked reduction (0.21 mM/min *vs* 0.17 mM/min). For the oxidation of isopropanol, the similar enzymological properties of *Ac*CR were found using NAD^+^ or NADP^+^ as cofactor. Furthermore, a broad range of ketones such as aryl ketones, α-ketoesters and aliphatic ketones could be enantioselectively reduced into the corresponding chiral alcohols by this enzyme with high activity.

## Introduction

Enantiopure alcohols are widely used as building blocks in pharmaceutical, agrochemical, flavor, and functional material industries. The “green” synthesis catalyzed by enzymes, microorganisms and plant cells for the production of various enantiopure alcohols has attracted considerable attention due to its high efficiency, high enantioselectivity, mild reaction condition, and low environmental pollution [1], [2]. Carbonyl reductase is one of the attractive biocatalysts for synthesis of enantiopure alcohols [3], and widely expressed in various organisms from microorganisms to mammals such as *Bacillus* [4], *Neurospora crassa* [5] and human brain [6]. Carbonyl reductases belong to the oxidoreductase family that often need cofactors such as NAD(H) or NADP(H) to be functionally active, which could be divided into three subtypes based on the coenzyme requirements: (1) NAD(H)-specific, like carbonyl reductase originated from *Candida viswanathii* [7]; (2) NADP(H)-specific, such as carbonyl reductase from *Neurospora crassa* [5]; (3) dual coenzyme specific, like carbonyl reductase from *Candida parapsilosis* [8].

The substrate specificity of an enzyme is largely determined by the structure of substrate-binding site and the general plasticity of this region. Most carbonyl reductases often have more than one type of substrates. For example, the carbonyl reductase from *Neurospora crassa* could catalyze asymmetric reduction of ketones, diketones, *α*-keto esters, and *β*-keto esters [5] and the carbonyl reductase from *Streptomyces coelicolor* could be used for synthesis of chiral alcohols from aryl ketones, *α*-keto esters, and *β*-keto esters with high enantioselectivity [9].

Obviously, carbonyl reductases with good catalytic efficiency and excellent stereoselectivity can improve the product yield and simplify the purification processes. Some carbonyl reductases with high enantioselectivity have been reported. For example, the carbonyl reductases from *Kluyveromyces thermotolerans* [10] and *Candida glabrata* [11] were able to catalyze the asymmetric reduction of methyl *o*-chlorobenzoylformate to methyl (*R*)-*o*-chloromandelate with high enantioselectivity (99% *e.e*.). Combined with the effective cofactor regeneration systems, the carbonyl reductases showed great potential for the industrial application.

In the present study, the discovery, purification, and characterization of a novel carbonyl reductase from *Acetobacter* sp. CCTCC M209061 (*Ac*CR) have been described. It was found that *Ac*CR was capable of catalyzing the anti-Prelog asymmetric reduction of various types of ketones with excellent enantioselectivity. Furthermore, the potential of *Ac*CR acted as a robust industrial biocatalyst was examined for efficient synthesis of several pharmaceutically important chiral alcohol intermediates.

## Materials and Methods

### Chemicals

2′-Methoxyacetophenone (99% purity), 3′-methoxyacetophenone (97% purity), 4′-methoxyacetophenone (99% purity), (*R*)-1-(4-fluorophenyl)ethanol (97% purity), (*R*)-1-(4-chlorophenyl)ethanol (95% purity), 4′-bromoacetophenone (98% purity), (*R*)-1-(4-bromophenyl)ethanol (95% purity), (*S*)-1-(4-bromophenyl)ethanol (95% purity), 4′-methylacetophenone(96% purity), methyl acetoacetate (99% purity), methyl (*R*)-3-hydroxybutyrate (99% purity), methyl (*S*)-3-hydroxybutyrate (99% purity), ethyl 3-hydroxybutyrate (98% purity), ethyl (*R*)-3-hydroxybutyrate (98% purity), 3,3-dimethyl-2-butanone (97% purity), 4-(trimethylsilyl)-3-butyn-2-one (97% purity), 4-(trimethylsilyl)-3-butyn-2-ol (97% purity), 1-(trimethylsilyl)ethanone (97% purity) and 1-(trimethylsilyl)ethanol (98% purity) were purchased from Sigma–Aldrich (St Louis, MO, USA). 1-(2-Methoxylphenyl) ethanol (99% purity), 1-(3-methoxylphenyl) ethanol (97% purity) and 1-(4-nitrophenyl))ethanol (95% purity), were purchased from J&K Scientific Ltd (Beijing, China). 1-(4-Methoxylphenyl)ethanol (95% purity), 4′-fluoroacetophenone (99% purity), 1-(4-fluorophenyl)ethanol (98% purity), 4′-chloroacetophenone (98% purity), 1-(4-chlorophenyl)ethanol (97% purity), 4′-nitroacetophenone (98% purity), 1-(4-methylphenyl)ethanol (97% purity), 3,3-dimethyl-2-butanol (99% purity), *n*-decane (99% purity), 2-pentanone (99% purity), 2-pentanol (99% purity), (*S*)-2-pentanol (97% purity), 2-octanone (98% purity), 2-octanol (98% purity) and (*R*)-2-octanol (99% purity) were purchased from Alfa Aesar (Ward Hill, MA, USA). Coenzymes, glucose-6-phosphate (G-6-P) and glucose-6-phosphate dehydrogenase (GPDH) were purchased from Qiyun Biotechnology Company (Guangzhou, China). All other reagents and solvents were of analytical grade and used without further purification.

### Microorganism and Growth

*Acetobacter* sp. CCTCC M209061 was previously isolated from Chinese kefir grains [12] and kept in our laboratory at −80°C. It was cultivated and harvested as described previously [13].

### Purification of Carbonyl Reductase

After harvesting, 10 g of wet cells were re-suspended in 30 ml buffer A (20 mM sodium phosphate, pH 6.5, 0.5 mM phenylmethanesulfonylfluoride, 1.0 mM *β*-mercaptoethanol, 0.5 mM MnSO_4_), and disintegrated by 40 min sonication at 4°C. Cell debris was removed by centrifugation (12,000×g) at 4°C for 15 min, and the supernatant (crude extract enzyme) was mixed with ammonium sulfate (30% saturation). After centrifugation (12,000×g at 4°C for 15 min), the concentration of ammonium sulfate in supernatant was increased to 80% saturation under which the crude enzyme was precipitated and collected by centrifugation (12,000×g) at 4°C for 15 min. After dialysis against buffer A overnight, the crude enzyme (around 70 mg protein) was loaded on a pre-equilibrated DEAE-Sepharose column (GE Healthcare, Uppsala, Sweden) and purified on an ÄKTA Purifier 100 system (GE Healthcare, Piscataway, NJ, USA) at 4°C. The elution was carried out with increasing concentration of NaCl (0, 0.1, 0.2, 0.5 and 1.0 M) in buffer B (20 mM sodium phosphate, pH 6.5, 0.5 mM MnSO_4_). The active fraction was eluted by buffer B with 0.2 M NaCl, pooled and concentrated by ultrafiltration with Centriprep YM-10 from Millipore (Bedford, MA, USA). Then Native-PAGE was performed according to the procedure reported by Chen et al [14]. The separating and stacking gels contained 9% and 5% acrylamide, respectively. The electrophoresis was performed at 4°C and the subsequent experiment was performed as described by Chen et al [14]. The target segment was chopped into small pieces, transferred into a dialysis bag with a cutoff molecular weight of 11 kDa and electrophoresed for 10 min to recollect the enzyme. The purified enzyme solution was subsequently desalted and concentrated by ultrafiltration using Centriprep YM-10.

### Molecular Mass Determination

The purified enzyme was loaded on a Superdex G-200 column (1.0 cm ×30 cm, GE Healthcare, Piscataway, NJ, USA) and the apparent molecular mass was determined by gel permeation chromatography with the mobile phase of phosphate buffer (50 mM, pH 7.0) containing 0.15 M NaCl at a flow rate of 0.2 ml/min. The column was pre-calibrated with standard molecular mass markers including ovalbumin (43.0 kDa), conalbumin (75.0 kDa), aldolase (158.0 kDa), ferritin (440.0 kDa), and thyroglobulin (669.0 kDa).

SDS-PAGE was conducted according to the procedure reported by Laemmli [15]. The subunit molecular mass was determined by the R_F_ values. The calibration curve was obtained using standard low molecular mass markers including lysozyme (14.3 kDa), trypsin inhibitor (20.1 kDa), carbonic anhydrase (29.0 kDa), ovalbumin (44.3 kDa), bovine serum albumin (66.4 kDa), and phosphorylase b (97.2 kDa).

### Enzyme Activity Assays

The initial rate of *Ac*CR-catalyzed reduction of ketone was determined by monitoring the change in absorbance at 340 nm over 1 min on a Shimadzu UV-3010 spectrophotometer (Shimadzu, Japan). The NADH- and NADPH-based reductions were carried out at 25°C in 0.25 ml phosphate-citrate buffer (50 mM, pH 5.0 or 7.5). One unit (U) of *Ac*CR was defined as the amount of enzyme that catalyzed the conversion of 1 *µ*mol NADH or NADPH per minute.

Since there were other NADH- and NADPH-oxidases in crude enzyme preparations (crude extract and ammonium sulfate precipitate), the change in the absorbance of NADH or NADPH at 340 nm might not be only caused by the *Ac*CR-mediated reduction reaction. Therefore, the conversion of 4′-chloroacetophenone was determined by gas chromatography (GC). Reactions were carried out with 30 mM NADH or NADPH and 5 mM 4′-chloroacetophenone at 25°C in 0.25 ml phosphate-citrate buffer (50 mM, pH 5.0 or 7.5). One unit (U) of *Ac*CR was defined as the amount of enzyme that catalyzed the conversion of 1 *µ*mol 4′-chloroacetophenone per min.

The NAD^+^- and NADP^+^-linked oxidations were conducted with NAD^+^ or NADP^+^ and isopropanol at 35°C in 0.25 ml of sodium phosphate buffer (50 mM, pH 8.0). One unit (U) of *Ac*CR was defined as the amount of enzyme that catalyzed the production of 1 *µ*mol NADH or NADPH per min.

Protein contents were determined by the method of Bradford [16].

### Effects of pH and Temperature

The pH dependence of enzyme activity was studied at various pHs (pH 4.0–9.5) using 50 mM citrate–phosphate (pH 4.0–8.0), Tris-HCl (pH 7.5–8.5) and glycine–NaOH (pH 8.6–9.5) as the buffer, respectively. The effect of temperature on enzyme activity was investigated at varied temperatures from 10 to 45°C. The pH stability of the purified enzyme was assessed by determining the enzyme activity after 5 days incubation at 4°C in buffers with various pHs. The thermal stability of the enzyme was assessed after 5 h incubation at varied temperatures from 15 to 40°C. The enzyme activity was measured as described above.

### Effects of Various Additives

The effects of metal ions, metal ion chelators, and thiol reagents on the catalytic activity of *Ac*CR were determined by pre-incubating the enzyme with various additives in phosphate-citrate buffer (50 mM, pH 5.0) at 25°C for 15 min. The enzyme activity was then measured as described above. The relative activity was calculated as the percentage of the activity of the enzyme observed without the examined additives.

### Substrate Specificity

The activity of *Ac*CR for each specific substrate was determined by the method described above using NADH as the cofactor. The relative activity towards 4′-chloroacetophenone was defined as 100%.

### Bioconversion of Various Ketones

The reductions of ketones were performed in a 10-ml Erlenmeyer flask with 2 ml phosphate-citrate buffer (50 mM, pH 5.0) containing 30 mM NADH and 5 mM ketones. The flask was capped with a septum and pre-incubated in an incubator with shaking at 25°C for 10 min. The purified *Ac*CR was added to initiate the reaction. Samples (50 *µ*l) were withdrawn at specified time intervals. The product and the residual substrate were extracted twice with 100 *µ*l isopropyl ether (aryl ketones) or *n*-hexane (other ketones) containing 5 mM *n*-decane (as an internal standard) prior to GC analysis.

### Kinetic Parameters Assays

The initial reaction rates were determined under the optimum conditions. For the reduction of 4′-chloroacetophenone, NADH or NADPH concentrations varied from 0.04 to 1.50 mM or 0.02 to 0.40 mM, and 4′-chloroacetophenone concentrations varied from 1.5 to 20.0 mM. For the oxidation of isopropanol, NAD^+^ and NADP^+^ concentrations varied from 0.2 to 20.0 mM, and isopropanol concentrations varied from 13 to 208 mM. All measurements were performed in triplicate. Michaelis-Menten or Hill equation was used to fit the data (initial reaction rate *vs*. substrate concentration), and the kinetic parameters of *Ac*CR-catalyzed reduction and oxidation reactions, K_m_ and V_max_ values, were obtained from the fit.

### Supply of Cofactors

The reactions were carried out in 0.25 ml of 50 mM phosphate-citrate buffer at pH 5.0 (NADH acted as cofactor) or pH 7.5 (NADPH acted as cofactor) containing 5 mM 4′-chloroacetophenone, 10 mM 2-octanone, or 15 mM ethyl acetoacetate. The cofactor was supplied by three methods: (1) direct addition of 30 mM NADH or NADPH; (2) addition of 30 mM G-6-P, 0.2 U/ml GPDH and 0.2 mM NADPH; (3) 130 mM isopropanol and 0.2 mM or 1 mM NADH/NADPH. Samples (50 *µ*l) were withdrawn at specified time intervals. The product and the residual substrate were extracted twice for GC analysis.

In addition, using G-6-P/GPDH system to regenerate NADPH, the reactions were carried out in 25 ml of sodium phosphate buffer (50 mM, pH 7.5). When the reaction was completed with no further product formation (2–4 h), the aqueous layer was extracted three times with 10 ml of isopropyl ether for 4′-chloroacetophenone or ethyl ester for 2-octanone and ethyl acetoacetate. The organic layers were combined and dried by Na_2_SO_4_, and analyzed by GC and Polarimeter SGW-2.

### Analytical Methods

The organic extracts of reaction mixtures were analyzed with a GC 2010 g from Shimadzu Corp. (Kyoto, Japan) equipped with chiral columns {HP Chiral 20% and CP-ChiraSil-DEX CB from J&W Scientific (Folsom, CA, USA) and HP Chiral 10% from Hewlett Packard (USA)} and a flame ionization detector. Nitrogen was used as the carrier gas. The split ratio was 1∶100 (v/v). The injector and the detector temperatures were both set at 250°C. The detailed methods were summarized in Table S1, and the *R*- or *S*-configuration of the formed product (chiral alcohol) was determined by comparison with the reference compound.

The [α]_D_ of the product was measured by Polarimeter SGW-2. The [α]_D_ of (*R*)-1-(4-chlorophenyl)ethanol, ethyl (*R*)-3-hydroxybutyrate, and (*R*)-2-octanol were [α]^24.3^_D_ +49.4° (C = 0.32, isopropyl ether), [α]^24.3^_D_ - 45.4° (C = 0.17, ethyl ester), and [α]^24.3^_D_ - 11.5° (C = 0.23, ethyl ester), respectively.

### Hunting for *Ac*CR gene sequence from genome of *Acetobacter* sp. CCTCC M209061

Genomic DNA of *Acetobacter* sp. CCTCC M209061 was extracted and purified using a bacterial genomic DNA Kit (Generay, Shanghai, China). Oligonucleotide primers were designed according to the published gene sequence of oxidoreductase from *Acetobacter pasteurianus* 386B (Sequence ID: ref|YP_008391729.1). Primer 1: 5′-ATGGCACGTGTAGCAGGCAAGGTT-3′; primer 2: 5′-TTATTGTGCAGTGTACCCACCATCAAT-3′. The DNA fragment of *Ac*CR was amplified by polymerase chain reaction (PCR) using the KOD DNA polymerase (TOYOBO. Osaka, Japan). The PCR amplification was performed with 2 min of pre-denaturation at 94°C, followed by 30 cycles of 15 s at 98°C, 30 s annealing at 64°C, 40 s extension at 68°C, and a final extension of 7 min at 68°C. The PCR products were analyzed and purified using 1.2% agarose gel. After electrophoresis, the amplified *Ac*CR DNA band was collected and sequenced. The obtained sequences were classified by comparison with those in public DNA databases.

## Results and Discussion

### Activity of Carbonyl Reductase over Cultivation Time

**Figure 1:**
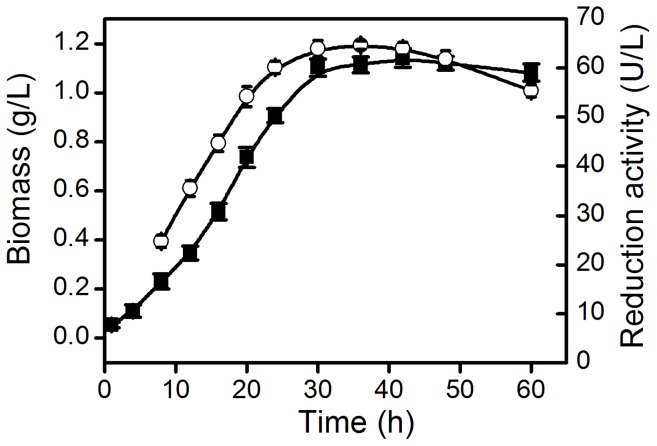
The biomass of *Acetobacter* sp. CCTCC M209061 and the reduction activity of *Ac*CR during its growing process. Biomass (▪); Reduction activity (○).

*Acetobacter* sp. CCTCC M209061 was cultivated and harvested under conditions reported previously [13]. The growth curve of *Acetobacter* sp. CCTCC M209061, and the dependence of the reduction activity of *Ac*CR from *Acetobacter* sp. CCTCC M209061 cells on cultivation time were illustrated in Figure 1. As can be seen, the biomass and the total activity of *Ac*CR remarkably increased with increasing cultivation time up to 30 h (growth phase). A further increase in cultivation time (beyond 30 h) resulted in a moderate fall in the total activity of *Ac*CR, but led to no significant increase in the biomass. Additionally, the specific activity of *Ac*CR (relative to the biomass) reached the highest value at about 15 h cultivation (mid-log phase) but leveled off afterwards. The total activity of *Ac*CR stayed at a high level (above 60 U/L) between 24 and 42 h of cultivation time. Interestingly, it was found that the storage stability of *Ac*CR from *Acetobacter* sp. CCTCC M209061 was dependent on the cultivation time of the cell. The activities of *Ac*CRs from the cells collected at different cultivation times were assessed after the storage for seven days at 4°C, and the enzyme *Ac*CR from the cells harvested between 24 and 36 h of cultivation time remained around 70–80% of its original activity, and the enzyme from the cells collected at less than 24 h or more than 36 h of cultivation time maintained approximately 50–60% of its initial activity. Obviously, the optimal cultivation time is 30 h for the reduction activity of *Ac*CR isolated and purified from *Acetobacter* sp. CCTCC M209061.

### Purification of Carbonyl Reductase

**Figure 2:**
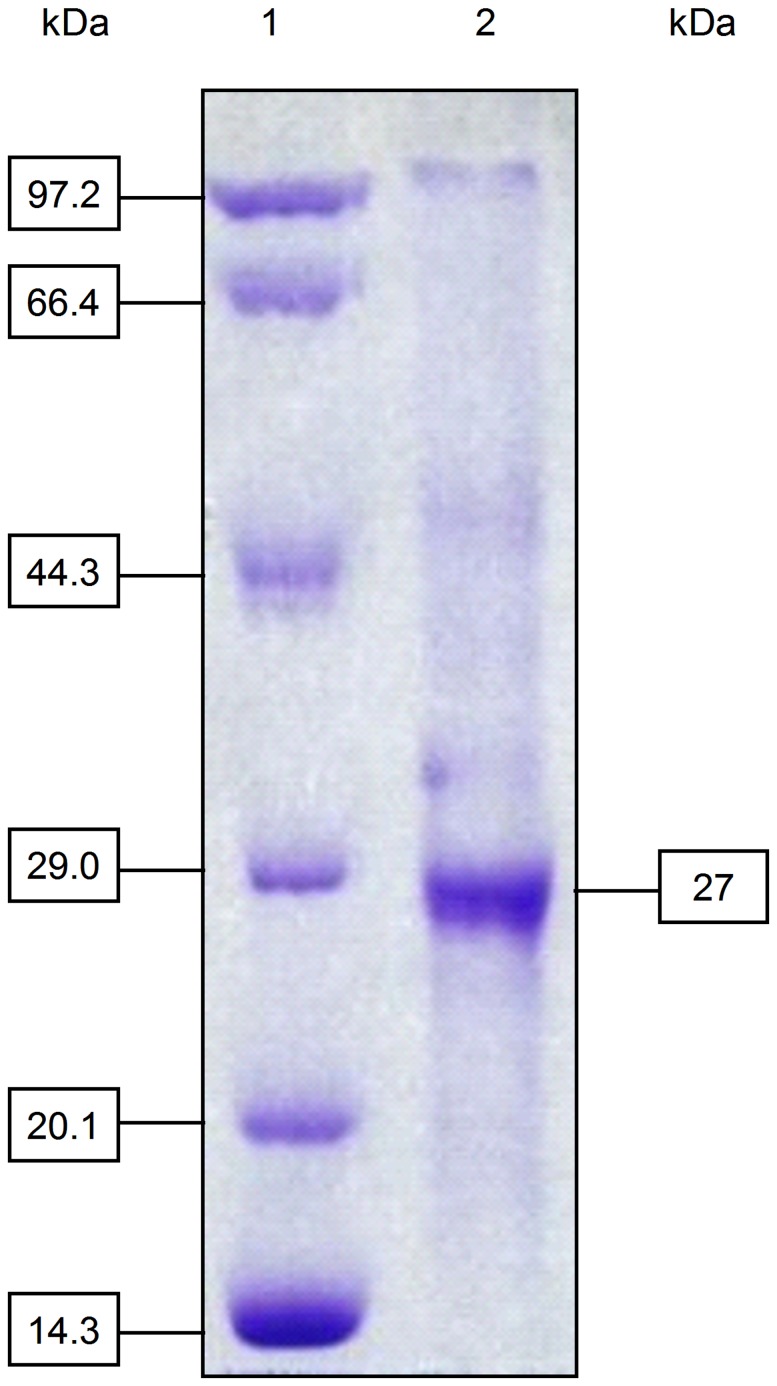
SDS-PAGE analysis of the purified *Ac*CR. Gel was stained with 0.05% Coomassie Blue R-250: lane 1, molecular weight markers; lane 2, the purified enzyme with a molecular mass of 27 kDa.

In our early attempts to purify the *Ac*CR from *Acetobacter* sp. CCTCC M209061 cells, the enzyme quickly lost its activity during ion exchange chromatography or Native-PAGE, and only about 40% of activity was obtained after the precipitation with ammonium sulphate. After many purification conditions were examined, surprisingly, the addition of 0.5 mM Mn^2+^ to the buffer was found to effectively prevent the enzyme *Ac*CR deactivation during the purification process, which was similar to that observed during the purification of (*R*)-phenylethanol dehydrogenase [17]. From the data for each purification step summarized in Table 1, the cell-free extract was precipitated with ammonium sulfate fractionation and the active enzyme protein was collected in the range of 30–80% saturation. This crude enzyme was then purified by the DEAE-Sepharose column. The active enzyme was detected in the eluent of 0.2 M NaCl with a 5.0-fold enrichment. The enzyme was concentrated and subsequently purified by Native-PAGE (Figure S1). After the final purification step, the *Ac*CR was purified by 27.5-fold to a specific activity of 3.85 U/mg-protein with a total yield of 0.4%. The apparent molecular mass of the native enzyme was estimated to be 104 kDa by gel filtration and the molecular mass of the subunit was estimated to be 27 kDa by SDS-PAGE (Figure 2), indicating that the enzyme *Ac*CR has a homotetrameric structure.

**Table 1:** Purification of *Ac*CR from *Acetobacter* sp. CCTCC M209061.

Purification step	Total protein (mg)	Total activity (U)	Specific activity (U/mg)	Yield (%)	Enrichment (fold)
Cell-free extract	757.8	105.9	0.14	100.0	1.0
(NH_4_)_2_SO_4_ precipitation	407.6	76.1	0.19	71.9	1.3
DEAE Sepharose	51.0	35.6	0.70	33.6	5.0
Native-PAGE	0.1	0.4	3.85	0.4	27.5

All operations were carried out at 4°C.

Cell-free extract and (NH_4_)_2_SO_4_ precipitation: 1 U =  reduction of 1 *µ*mol 4′-chloroacetophenone per min.

DEAE Sepharose and Native-PAGE: 1 U =  oxidation of 1 *µ*mol NADPH per min.

### Effect of Temperature on the Activity and Stability of *Ac*CR

**Figure 3:**
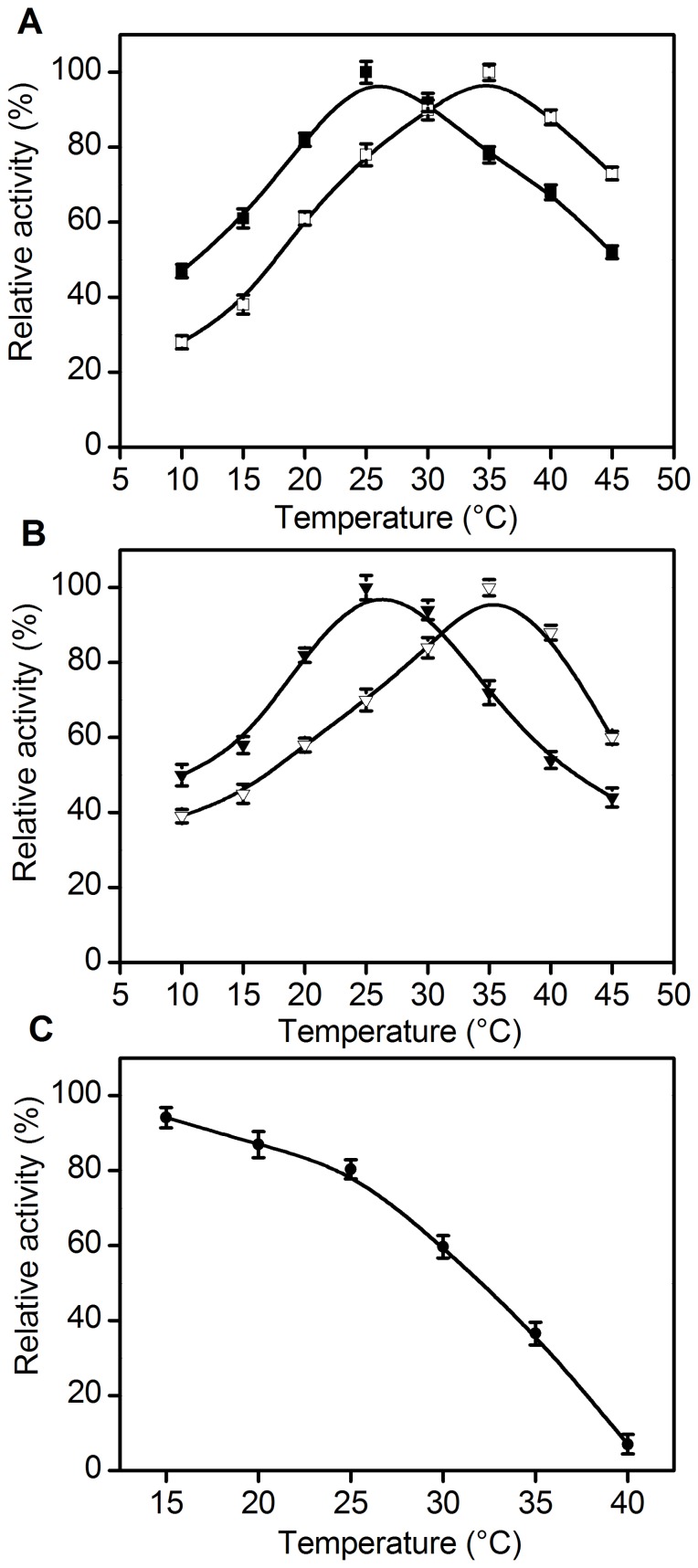
Relative activity of *Ac*CR at different temperatures. (A) Reduction of 4′-chloroacetophenone using NADPH as cofactor (▪); Oxidation of isopropanol using NADP^+^ as cofactor (□); (B) Reduction of 4′-chloroacetophenone using NADH as cofactor (▾); Oxidation of isopropanol using NAD^+^ as cofactor (□); The activity was measured in the temperature range of 10–45°C. The pH value was 5.0 (NADH as cofactor) or 7.5 (NADPH as cofactor) for reduction of 4′-chloroacetophenone or 8.0 for oxidation of isopropanol, respectively. The relative activity of *Ac*CR at each optimal temperature for reduction and oxidation reactions was defined as 100%. (C) Thermal stability (•). The residual activity of *Ac*CR after 5 h incubation (buffer pH 6.5) at varying temperatures were measured at 25°C.

*Ac*CR was found to be a dual coenzyme specific enzyme and showed reduction and oxidation activities in the presence of either NAD(H) or NADP(H). This might be due to its unique structure that allows dual coenzyme binding. Initially, the effects of various temperatures on the activity of *Ac*CR was investigated for both reduction and oxidation reactions. The optimal temperature was found to be 25°C, for reduction of 4′-chloroacetophenone when NADPH (Figure 3A) or NADH (Figure 3B) was used as cofactor, which was similar to that observed with carbonyl reductase from *G. capitatum* JCM 3908 [18], but was much lower than that with carbonyl reductases from *Thermus thermophilus* HB27 (85°C) [19] and *Geotrichum candidum* (65°C) [20]. The maximum activity for oxidation of isopropanol was observed at 35°C using NADP^+^ (Figure 3A) or NAD^+^ (Figure 3B) as cofactor.

The thermal stability of *Ac*CR was determined at varying temperatures from 15 to 40°C (Figure 3C). The enzyme was very sensitive to reaction temperature and lost its activity quickly when temperature was higher than 35°C. The enzyme retained more than 80% of its original activity after 5 h incubation at a temperature range of 15–25°C. However, a significant drop in *Ac*CR's activity was observed when reaction temperature was raised to 30°C, and *Ac*CR remained only about 7% of its initial activity after 5 h incubation at 40°C. Obviously, the thermal stability of *Ac*CR was not very satisfactory. Protein engineering strategy might be promising approach to further improve the activity and stability of the enzyme *Ac*CR [21].

### Effect of pH on the Activity and Stability of *Ac*CR

**Figure 4:**
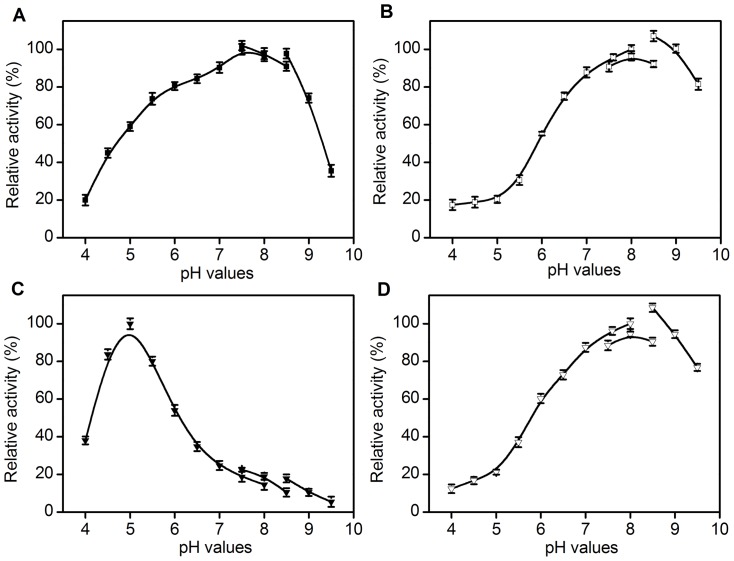
Relative activity of *Ac*CR at different pH values. (A) Reduction of 4′-chloroacetophenone using NADPH as cofactor (▪); (B) Oxidation of isopropanol using NADP^+^ as cofactor (□); (C) Reduction of 4′-chloroacetophenone using NADH as cofactor (▾); (D) Oxidation of isopropanol using NAD^+^ as cofactor (□). The buffers used were 50 mM citrate-phosphate (pH 4.0–8.0), Tris-HCl (pH 7.5–8.5) and glycine–NaOH (pH 8.6–9.5) buffers. The enzyme's activity was measured in the pH range of 4.0–9.5 at 25°C for reduction of 4′-chloroacetophenone and at 35°C for oxidation of isopropanol. The relative activity of *Ac*CR at each optimal pH for reduction and oxidation reactions was defined as 100%.

The effect of pH on the activity of *Ac*CR was examined for both reduction and oxidation at a range of pH 4.0–9.5 (Figure 4). *Ac*CR manifested the maximum reduction activity towards of 4′-chloroacetophenone using NADPH (Figure 4A) and NADH (Figure 4C) as cofactors at pH 7.5 and pH 5.0, respectively. The optimal pH for oxidation of isopropanol was found to be pH 8.0 when either NADP^+^ (Figures 4B) or NAD^+^ (Figures 4D) was acted as cofactor. Interestingly, it was noted that *Ac*CR showed significantly different activities in the used three buffers at the same pH value (pH 7.5, 8.0 or 8.5) (Figure 4), which might be mainly attributable to the influences of the ionic strengths and types of these buffers. The detailed reasons for this unexpected observation need further investigations and are underway in our laboratory. When NADPH and NADP^+^ were used as cofactors, the optimal pH values for reduction and oxidation reactions with *Ac*CR were very close (7.5 and 8.0), which was similar to that with alcohol dehydrogenase from *Lactobacillus kefir* [22]. When NADH and NAD^+^ were used as cofactors, on the contrary, the optimal pH values for reduction and oxidation indicated great difference (5.0 and 8.0), which was in good accordance with the observation with alcohol dehydrogenase from *Candida maris* IFO10003 [22]. Additionally, in the case of *Ac*CR-catalyzed oxidation of isopropanol, both NADP^+^ and NAD^+^ as cofactors gave the same optimal pH value (pH 8.0), which was similar to that with isocitrate dehydrogenase from *Rhodomicrobium vannielii* [23].

**Figure 5:**
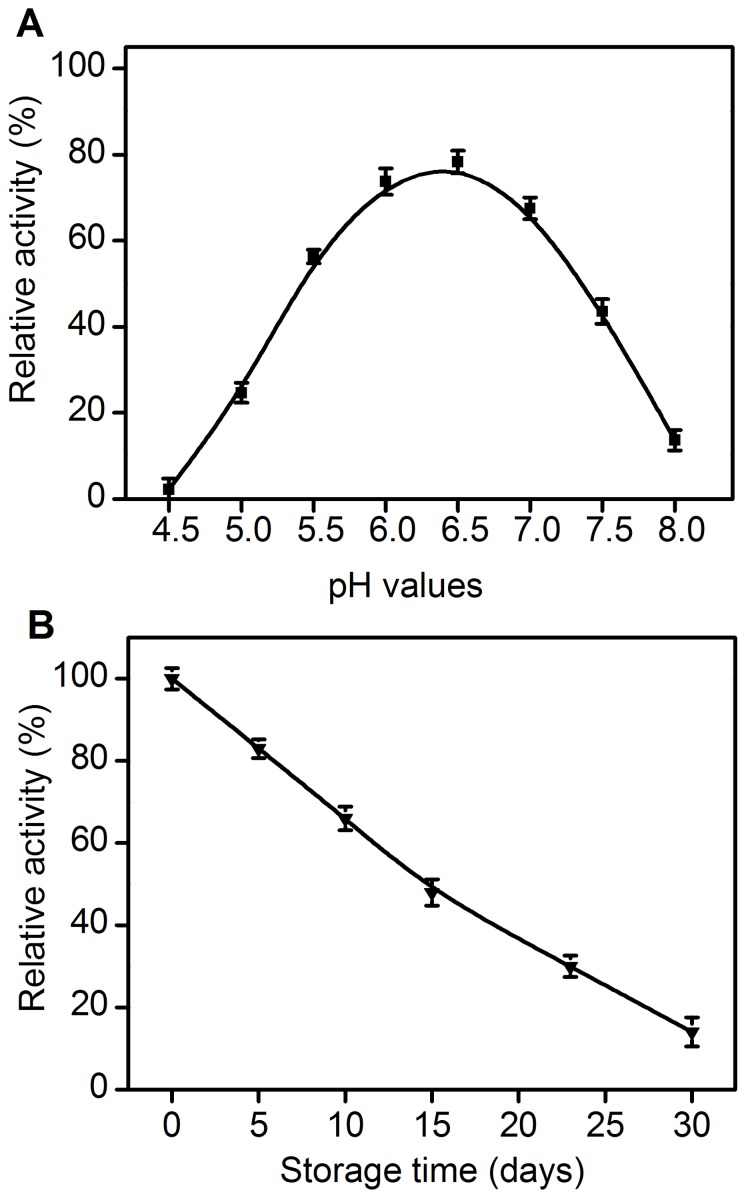
The stability of *Ac*CR. (A) pH stability (▪); The residual activities after incubation at various pH values (pH 4.5–8.0) for 5 days at 4°C were measured at buffer pH 5.0 and 25°C (NADH as cofactor). (B) Storage stability (▾). The residual activities after incubation at pH 6.5 for 30 days at 4°C were measured at buffer pH 5.0 and 25°C (NADH as cofactor). The relative activity of *Ac*CR without incubation was defined as 100%.

The pH stability of *Ac*CR was investigated by incubation in different buffers at varied pH values between 4.5 and 8.0 at 4°C (Figure 5A). The enzyme still retained around 80% of its initial activity after incubation for five days at pH 6.5. However, a considerable loss in activity was observed over a five-day period at pH values lower than 5.5 or higher than 7.0. Furthermore, the enzyme lost its total activity after being incubated in buffers with pH ≤4.5 or pH ≥8.0 for a same period.

### Storage Stability of *Ac*CR

The storage stability of the enzyme was investigated in sodium phosphate buffer (50 mM, pH 6.5) at 4°C. The purified *Ac*CR showed the unsatisfactory storage stability, which was supported by the observation that the enzyme lost about 17% and 50% of activity after storage for five days and two weeks, respectively (Figure 5B). Clearly, the *Ac*CR purified from *Acetobacter* sp. CCTCC M209061 cells was less stable than immobilized *Acetobacter* sp. CCTCC M209061 cells. Therefore, it is of great interest to enhance the stability (including storage stability) of *Ac*CR by immobilization in our further studies.

### Effects of Metal ions, Metal ion Chelators, and Thiol Reagents

It is well known that additives including metal ions have remarkable effects on the activity of carbonyl reductases from different sources. For example, Ca^2+^, Mn^2+^ and Mg^2+^ activated carbonyl reductase from *Leifsonia xyli* [24] and Zn^2+^ strongly inhibited carbonyl reductases from *Candida viswanathii* MTCC 5158 [7] and *Geotrichum candidum* [20]. So it is of great significance to explore the effects of various additives (metal ions, metal ion chelators, and thiol reagents) on the activity of *Ac*CR using reduction of 4′-chloroacetophenone as a model reaction. As evident from the data summarized in Table 2, Cu^2+^ (2 mM) showed inhibitory effect on *Ac*CR with 74% loss of activity, and Hg^2+^ and Ag^+^ inhibited completely the activity of *Ac*CR. Other metal ions tested exhibited positive effects on the enzyme's activity. Zn^2+^ and Ca^2+^ slightly increased the activity, and Mn^2+^, Ni^2+^ and Fe^2+^ greatly activated the enzyme, which was consistent with our previous observation that the activity of *Acetobaceter* sp. CCTCC M209061 cell was remarkably improved in the presence of Mn^2+^ during its cultivation [13]. Also, Co^2+^ and Mg^2+^ manifested significant activation to the enzyme. Thiol-binding reagent iodoacetamide had only weak effect on the enzyme's activity, suggesting that there were no essential -SH groups at the catalytic sites of *Ac*CR. *β*-Mercaptoethanol with a high concentration (40 mM) exhibited no significant effect on the enzyme's activity, indicating there were also no significant disulfide linkages present in the catalytic groups of the enzyme. Moreover, chelating agent EDTA had a strong inhibitory effect on the enzyme, indicating that metal ions were required for the enzyme's activity. Various surfactants such as SDS, Tween 80, and Triton X-100 were also examined for their effects on *Ac*CR's activity. All these detergents inhibited the enzyme's activity, which might be attributable to the disruption of hydrophobic interactions and enhancement in internal repulsive forces [25].

**Table 2:** Effects of various additives on the activity of *Ac*CR.

Additives	Concentration (mM)	Relative activity (%)
Control	0 mM	100
Mn^2+^	2 mM	319.9±3.6
	5 mM	334.2±4.2
Mg^2+^	2 mM	172.4±2.8
	5 mM	233.4±3.4
Ni^2+^	2 mM	286.9±4.6
	5 mM	315.3±5.9
Co^2+^	2 mM	201.4±5.3
	5 mM	232.9±1.9
Fe^2+^	2 mM	271.4±5.2
	5 mM	253.0±4.5
Ca^2+^	2 mM	115.8±4.7
	5 mM	135.7±3.9
Cu^2+^	2 mM	36.0±1.5
	5 mM	23.5±0.6
Zn^2+^	2 mM	141.5±1.9
	5 mM	131.3±1.0
Hg^2+^	2 mM	0
	5 mM	0
Ag^+^	2 mM	0
	5 mM	0
EDTA	2 mM	14.0±0.3
	5 mM	0
2-Mercaptoethanol	20 mM	99.5±1.5
	40 mM	97.1±1.7
Iodoacetamide	20 mM	93.0±2.5
	40 mM	70.3±2.7
SDS	2 mM	91.1±2.5
	5 mM	69.2±1.3
Tween 80	15 mM	49.3±1.1
	30 mM	28.8±0.7
Triton X-100	30 mM	30.5±1.1

The reduction activity of *Ac*CR was measured after being incubated at 25°C (NADH as cofactor) for 5 min with various additives in 50 mM phosphate-citrate buffer (pH 5.0). The relative activity was calculated by setting the activity without additive as 100%.

Obviously, the activity of *Ac*CR was strongly inhibited by some heavy metal ions, metal ion chelating reagents and protein detergents, and also effectively activated by specific metal ions such as Mn^2+^. Moreover, the above-described results clearly showed that there were no essential thiol groups or disulfide linkages at or near the catalytic sites of *Ac*CR.

### Substrate Specificity and Enantioselectivity of *Ac*CR

Substrate specificity and enantioselectivity are important characteristics of enzyme. Therefore, it was of great interest to investigate the substrate specificity and enantioselectivity of the novel *Ac*CR purified from *Acetobacter* sp. CCTCC M209061 cells. A broad range of carbonyl compounds (including 4′-chloroacetophenone) such as aryl ketones, α-ketoesters and aliphatic ketones were examined as the substrates for *Ac*CR. Initially, both NADH and NADPH were used as cofactors for *Ac*CR-catalyzed reductions of carbonyl compounds. The reaction rates and yields were measured at buffer pH 5.0 for NADH and buffer pH 7.5 for NADPH, respectively. The obtained yield and reaction rate with NADH as cofactor were clearly superior to those with NADPH. The higher V_max_ value was observed in the presence of 30 mM NADH. Thus, NADH was more suitable cofactor for *Ac*CR-catalyzed reduction than NADPH.

**Figure 6:**
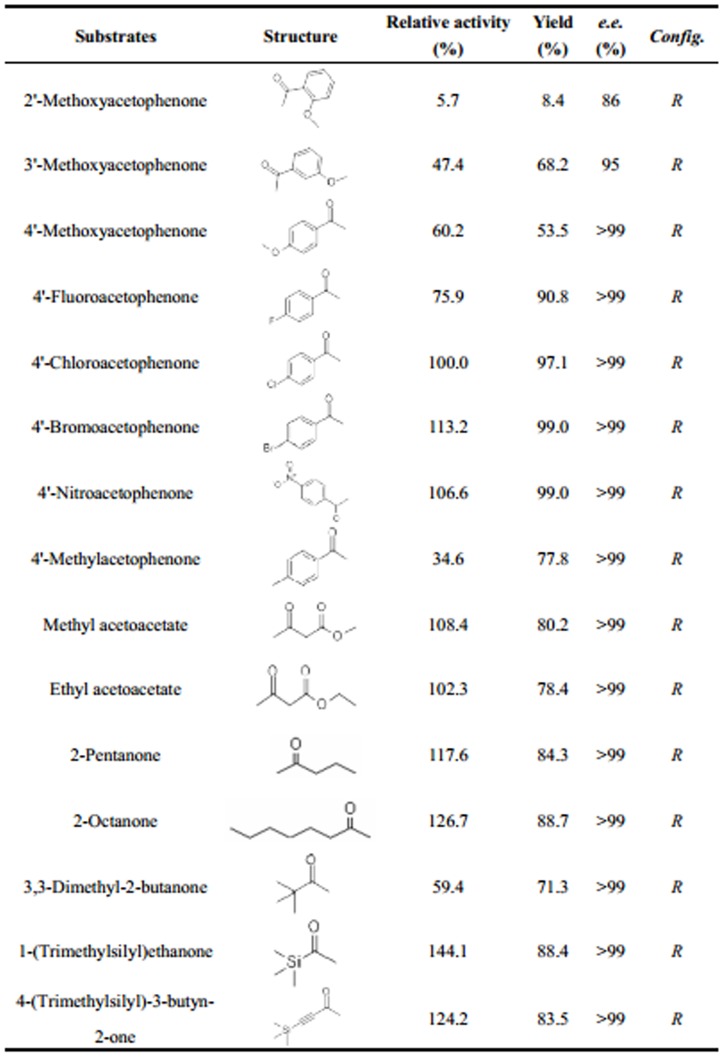
Effects of different substrates on AcCR-catalyzed asymmetric reduction of prochiral ketones.

As shown in Figure 6, NADH was used as cofactor and the relative activity of *Ac*CR for reduction of model substrate 4′-chloroacetophenone was defined as 100%. High product yield and excellent enantioselectivity (>99%) were achieved when the 4′-position of acetophenone was substituted by electron-withdrawing groups such as -Br, whereas the electron-releasing groups like -methyl had negative effects. Moreover, the enzyme did not perform well with 2′- and 3′- substituted acetophenone such as 2′-methoxyacetophenone (5.7%) and 3′-methoxyacetophenone (47.4%), which might be affected by steric hindrance [7]. Specifically, α-ketoesters, e.g. methyl acetoacetate and ethyl acetoacetate, whose chiral alcohol derivatives were potential building blocks for the synthesis of (+)-decarestrictine [26], were successfully converted with high yields (80.2% and 78.4%) and excellent enantioselectivity (>99%). Among the aliphatic ketones, 2-pentanone and 2-octanone were converted with a high yield and enantioselectivity. Interestingly, the enzyme seemed more active towards silicon-containing ketones than the natural ketones. Relative activities above 100% were achieved even though the reaction conditions were not optimized for each specific substrate. These results could be attributable to specific properties of the silicon atom, such as its larger atomic radius and smaller electronegativity compared with the carbon atom. Indeed, the replacement of certain specific carbon atoms by silicon makes chemical and physical characteristics of silicon-contained molecules different from those of conventional organic compounds [27].

*Ac*CR was capable of catalyzing the anti-Prelog [28] enantioselective reductions of all the tested substrates to the corresponding (*R*)-alcohols. Except for 2′-methoxyacetophenone, the enzyme exhibited high enantioselectivity towards these carbonyl compounds. Compared with other carbonyl reductases, the substrate specificity of *Ac*CR was similar to that of carbonyl reductase from *Leifsonia xyli* HS0904 [24] and very different from that of the enzyme from *Pichia stipitis* [29].

### Kinetic Study

The kinetic parameters of *Ac*CR for 4′-chloroacetophenone reduction and isopropanol oxidation were determined by measuring initial reaction rates with varying concentrations of substrates, and the obtained results were listed in Table 3. By analyzing of the kinetic data with Michaelis-Menten model and Hill model, it could be clearly seen that there was no cooperativity of the enzyme for these substrates except that NADH was used as the coenzyme for reduction of 4′-chloroacetophenone. The dependence of *Ac*CR's activity on concentrations of substrates used and the corresponding Michaelis-Menten curve or Hill curve were illustrated in Figures S2–5.

**Table 3:** Kinetic parameters of *Ac*CR for reduction of 4′-chloroacetophenone and oxidation of isopropanol.

Substrates	K_m_ c (mM)	V_max (_mM/min)
NADH	0.66±0.021	0.21±0.008
4′-Chloroacetophenone^a^	4.13±0.157^c^	--
NADPH	0.026±0.001	0.17±0.005
4′-Chloroacetophenone^b^	2.96±0.087	--
NAD^+^	1.33±0.045	0.069±0.003
Isopropanol^a^	48.32±0.986	--
NADP^+^	1.12±0.035	0.074±0.004
Isopropanol^b^	67.82±2.321	--

Kinetic constants were spectrophotometrically analyzed by determining the initial reaction rate at 340 nm at different substrate concentrations. Reactions were carried out in the optimal conditions. The dependence of *Ac*CR's activity on substrate concentrations and the corresponding Michaelis–Menten curve or Hill curve were illustrated in Figures S2–5.

a Determination for the NAD(H)-linked reaction.

b Determination for the NADP(H)-linked reaction.

c The apparent K_m_ value was considered as S_0.5_.

For the reduction of 4′-chloroacetophenone, the K_m_ value for NADH (0.66 mM) was over 25-fold greater than that for NADPH (0.026 mM), showing that the enzyme had a much higher affinity for NADPH than for NADH when the cofactors were at relatively low concentration levels, which was similar with the carbonyl reductase from *Candida parapsilosis* with dual cofactor specificity and strong preference for NADPH [30]. In contrast, some reductases preferred NADH such as the carbonyl reductase from *Pichia stipitis* [29]. The cofactor preferences exhibited by carbonyl reductases might be attributed to the electrostatic environment surrounding the 2′-hydroxyl (or phosphate) group of the adenosine ribose moiety of NADH (or NADPH) [31]. When NADH was used as cofactor, the response of *Ac*CR's activity to increasing concentration of 4′-chloroacetophenone was clearly sigmoidal with a Hill coefficient of 3.1, suggesting that the enzyme might possess four substrate-binding sites cooperated with each other when NADH was present. Similar substrate binding cooperativity was observed with the matrix isocitrate dehydrogenase from potato mitochondria when the response of enzyme activity to isocitrate concentration was studied, while the dependence of initial velocity on the NAD^+^ concentration indicated that there was no cooperativity between subunits [32]. The apparent K_m_ value (S_0.5_) for 4′-chloroacetophenone was 4.13 mM, which was also higher than 4′-chloroacetophenone K_m_ (2.96 mM) using NADPH as cofactor. These results showed that the enzyme preferred NADPH when catalyzing reduction of 4′-chloroacetophenone. However, it is interesting that the V_max_ value for NADH-based reduction of 4′-chloroacetophenone was slightly higher than that for NADPH-based reduction (0.21 mM/min *vs* 0.17 mM/min). The reasons for this result are not clear and are the subject of ongoing investigations in our research group.

For the oxidation of isopropanol, the K_m_ value for NAD^+^ (1.33 mM) and NADP^+^ (1.12 mM) were similar and higher than that for NADH and NADPH (0.66 mM and 0.026 mM). The K_m_ values for isopropanol were 48.32 mM (NAD^+^ acted as cofactor) and 67.82 mM (NADP^+^ acted as cofactor), which were also higher than that for 4′-chloroacetophenone (4.13 mM and 2.96 mM). The V_max_ values for oxidation of isopropanol (0.069 mM/min and 0.074 mM/min) were lower than those for reduction of ketones (0.21 mM/min and 0.17 mM/min). All of these results indicated that the enzyme had a clear preference for reduction over oxidation (Table 3).

### Coenzyme Supply for the Reduction of Ketones

For *Ac*CR, expensive coenzymes (NADH or NADPH) were required for asymmetric reduction of prochiral ketones. Three different methods for coenzyme supply were tested and compared: (1) direct addition of NADH or NADPH; (2) regeneration of NADPH by adding G-6-P and GPDH; (3) addition of isopropanol. In the case of the second method, about 0.2 U/ml GPDH was used to keep 1/1 ratio of the two enzymes.

As can be seen in Table 4, *Ac*CR gave different yields using the three cofactor supply methods. Although direct addition of NADH/NADPH could effectively prompt the reduction of ketones with high yields of 75–97%, it increased the cost for large-scale industrial applications because of the very expensive price of NADH or NADPH [33].

**Table 4:** Effects of coenzyme supply methods on *Ac*CR-mediated asymmetric reduction of prochiral ketones.

Coenzyme supply	Yield *(%)* (*R*)-1-(4-chlorophenyl)ethanol			Yield (%) (*R*)-2-octanol			Yield *(%)* (*R*)-3-hydroxybutyrate		
	30 min	60 min	120 min	30 min	60 min	120 min	30 min	60 min	120 min
30 mM NADH	84.1±2.8	95.5±2.5	97.1±3.3	66.8±2.5	82.0±3.1	88.7±2.6	29.2±1.1	49.7±1.7	78.4±1.8
30 mM NADPH	78.4±2.4	87.3±3.2	96.2±2.6	61.2±1.4	74.9±1.9	86.6±2.8	27.8±1.0	45.3±1.4	75.5±2.5
G-6-P/GPDH +0.2 mM NADPH	80.6±3.1	98.3±2.4	97.6±2.8	43.4±1.2	60.6±2.2	96.0±2.4	29.6±0.9	58.3±2.1	84.2±2.7
Isopropanol +0.2 mM NADH	7.5±0.2	11.3±0.3	19.7±0.7	4.5±0.1	7.6±0.2	13.4±0.2	3.4±0.1	4.5±0.1	7.2±0.2
Isopropanol +0.2 mM NADPH	8.9±0.3	14.5±0.5	24.6±0.8	5.8±0.2	9.2±0.2	16.3±0.3	4.6±0.2	6.1±0.2	9.8±0.3
Isopropanol +1 mM NADH	32.6±1.2	47.2±1.5	81.0±2.1	21.7±0.7	32.2±0.9	53.1±1.7	10.4±0.3	20.2±0.6	29.8±1.1
Isopropanol +1 mM NADPH	33.7±1.1	48.3±1.6	84.2±3.2	22.6±0.5	31.5±1.2	55.6±1.5	12.8±0.4	22.5±0.9	32.6±0.9

The reactions were performed in phosphate-citrate buffer (50 mM, pH 5.0 or 7.5) containing substrates (5 mM 4′-chloroacetophenone, 10 mM 2-octanone or 15 mM ethyl acetoacetate) and different cofactor supply systems at 25°C.

G-6-P: Glucose-6- phosphate.

GPDH: Glucose-6- phosphate dehydrogenase.

The highest yields (84–98%) were obtained by using the cost-efficient NADPH-regenerating system (G-6-P/GPDH), which has been widely used in enzymatic synthesis of chiral alcohols [34]. Clearly, this NADPH-regenerating system surpassed the direct addition of coenzymes.

The *in situ* regeneration of cofactors can be also achieved using isopropanol without addition of GPDH. The *Ac*CR-catalyzed oxidation of isopropanol can produce the reduced form of coenzymes, which in turn is used as cofactor for reduction of ketones. This method might be less expensive for cofactor supply and has been effectively used in many organic synthesis [17]. For the *in situ* regeneration of cofactors with addition of isopropanol, the achieved yields (30–85%) by together adding 1 mM NADH/NADPH to the reaction system were much higher than those with 0.2 mM NADH/NADPH.

In comparison with carbonyl reductases from other microorganisms such as *Leifsonia xyli* HS0904 [24], *Candida viswanathii* MTCC 5158 [7], *Geotrichum candidum* [20] and *Pichia stipitis* [29], *Ac*CR from *Acetobacter* sp. CCTCC M209061 showed unique biochemical properties (dual coenzyme, cooperativity) and excellent anti-Prelog enantioslectivity in asymmetric reduction of a broad range of ketones, and had tremendous potential for synthesis of enantiopure alcohols.

### Cloning of *Ac*CR gene

**Figure 7:**
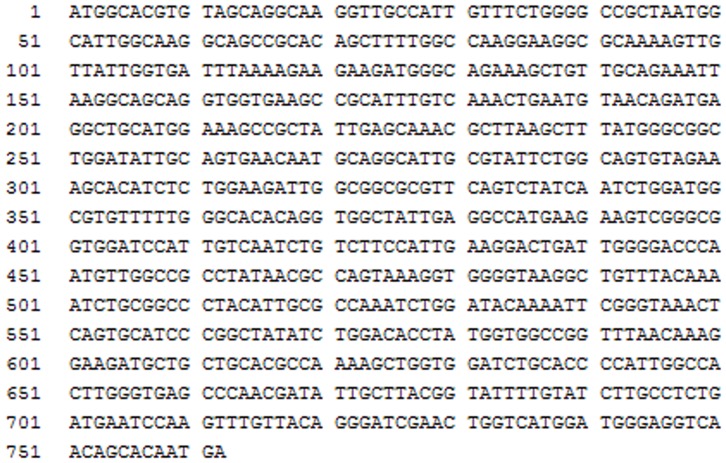
The gene sequences of *Ac*CR from *Acetobacter* sp. CCTCC M209061.

A genome mining approach was used for hunting *Ac*CR gene sequence according to the similar reports [35], [36]. The apparent molecular mass of the native *Ac*CR was estimated to be 104 kDa by gel filtration and the molecular mass of the subunit was estimated to be 27 kDa by SDS-PAGE (Figure 2), suggesting that the enzyme has a homotetrameric structure and similar catalytic properties to that of alcohol dehydrogenase from *Lactobacillus kefir* and *Lactobacillus brevis* [37]. Thus, by searching alcohol dehydrogenase from *Lactobacillus brevis* with GENBANK, it was found that an oxidoreductase from *Acetobacter pasteurianus* 386B (Sequence ID: ref|YP_008391729.1) had 51% sequence similarity with alcohol dehydrogenase from *Lactobacillus brevis*. The corresponding gene sequence of oxidoreductase from *Acetobacter pasteurianus* 386B (NC_021991.1) was found according to its amino acid sequence. Consequently, the primers were designed for hunting *Ac*CR gene sequence from *Acetobacter* sp. CCTCC M209061. A DNA fragment of 762 bp was obtained (Figure S6), and had 98% sequence similarity with the gene sequence of oxidoreductase from *Acetobacter pasteurianus* 386B (Figure S7). Thus, we successfully got the gene sequence of the novel *Ac*CR from *Acetobacter* sp. CCTCC M209061 (Figure 7).

## Conclusion

The novel *Ac*CR was purified from cytoplasm of *Acetobacter* sp. CCTCC M209061 and found to be a dual coenzyme specific enzyme, showing unique characteristics in reduction/oxidation reactions. Kinetic study indicated that the K_m_ and V_max_ values with NADH were higher than those with NADPH. The high anti-Prelog enantioselectivity of *Ac*CR in catalyzing reduction of various ketones highlighted its application potential for synthesis of chiral alcohols. The efficiency of the biocatalytic process could be further improved by co-expression of *Ac*CR and GPDH in a heterologous host.

## Supporting Information

Figure S1 **Clear Native-PAGE for purification of** ***Ac*** **CR.** (A) Crude enzyme after purification with DEAE-Sepharose; (B) *Ac*CR. The separating and stacking gels contained 9% and 5% acrylamide, respectively. The electrophoresis was performed at 4°C. The target segment (A) was chopped into small pieces, transferred into a dialysis bag with a cutoff molecular weight of 11 kDa and electrophoresed for 10 min to recollect the enzyme. The purified *Ac*CR was subsequently desalted and concentrated by ultrafiltration using Centriprep YM-10, which showed a single band by Native-PAGE (B). Gel was stained with 0.05% Coomassie Blue R-250.
(TIF)

Figure S2 **Dependence of** ***Ac*** **CR's activity on concentrations of NADH (A) and 4′-chloroacetophenone (B).** Substrate saturation curves were shown with Lineweaver-Burk plot (A) and Hill plot (B) inset. The enzyme's activity was estimated under standard assay conditions, and NADH concentrations varied from 0.04 to 1.50 mM (4′-chloroacetophenone concentration was fixed at 5 mM) and 4′-chloroacetophenone concentrations varied from 1.5 to 20.0 mM (NADH concentration was fixed at 1 mM). The kinetic parameters, K_m_ and V_max_ values, were graphically determined from the Lineweaver-Burk and Hill plotting.
(TIF)

Figure S3 **Dependence of** ***Ac*** **CR's activity on concentrations of NADPH (A) and 4′-chloroacetophenone (B).** Michaelis–Menten curves were shown with Lineweaver-Burk plots inset. The enzyme's activity was estimated under standard assay conditions, and NADPH concentrations varied from 0.02 to 0.40 mM (4′-chloroacetophenone concentration was fixed at 5 mM) and 4′-chloroacetophenone concentrations varied from 1.5 to 20.0 mM (NADPH concentration was fixed at 0.1 mM). The kinetic parameters, K_m_ and V_max_ values, were graphically determined from the Lineweaver-Burk plotting.
(TIF)

Figure S4 **Dependence of** ***Ac*** **CR's activity on concentrations of NAD^+^ (A) and isopropanol (B).** Michaelis–Menten curves were shown with Lineweaver-Burk plots inset. The enzyme's activity was estimated under standard assay conditions, and NAD^+^ concentrations varied from 0.2 to 20.0 mM (isopropanol concentration was fixed at 110 mM) and isopropanol concentrations varied from 13 to 208 mM (NAD^+^ concentration was fixed at 5 mM). The kinetic parameters, K_m_ and V_max_ values, were graphically determined from the Lineweaver-Burk plotting.
(TIF)

Figure S5 **Dependence of** ***Ac*** **CR's activity on concentrations of NADP^+^ (A) and isopropanol (B).** Michaelis–Menten curves were shown with Lineweaver-Burk plots inset. The enzyme's activity was estimated under standard assay conditions, and NADP^+^ concentrations varied from 0.2 to 20.0 mM (isopropanol concentration was fixed at 110 mM) and isopropanol concentrations varied from 13 to 208 mM (NADP^+^ concentration was fixed at 5 mM). The kinetic parameters, K_m_ and V_max_ values, were graphically determined from the Lineweaver-Burk plotting.
(TIF)

Figure S6 **PCR amplification of** ***Ac*** **CR gene sequence.** Lane A: 10 *µ*l sample, lane M: marker.
(TIF)

Figure S7 **Comparative analysis gene sequences of oxidoreductase from** ***Acetobacter pasteurianus*** **386B and** ***Ac*** **CR from** ***Acetobacter*** **sp. CCTCC M209061.** A: gene sequence of oxidoreductase from *Acetobacter pasteurianus* 386B; B: gene sequence of *Ac*CR from *Acetobacter* sp. CCTCC M209061.
(TIF)

Table S1 **GC analytic methods for various carbonyl compounds and their corresponding chiral alcohols.**
(DOC)
