# Supplementary figures and images for: A Novel Carbonyl Reductase with Anti-Prelog Stereospecificity from Acetobacter sp. CCTCC M209061: Purification and Characterization
Source: PLoS One. 2014 Apr 16;9(4):e94543. doi: 10.1371/journal.pone.0094543 (PMC3989197; doi:10.1371/journal.pone.0094543)

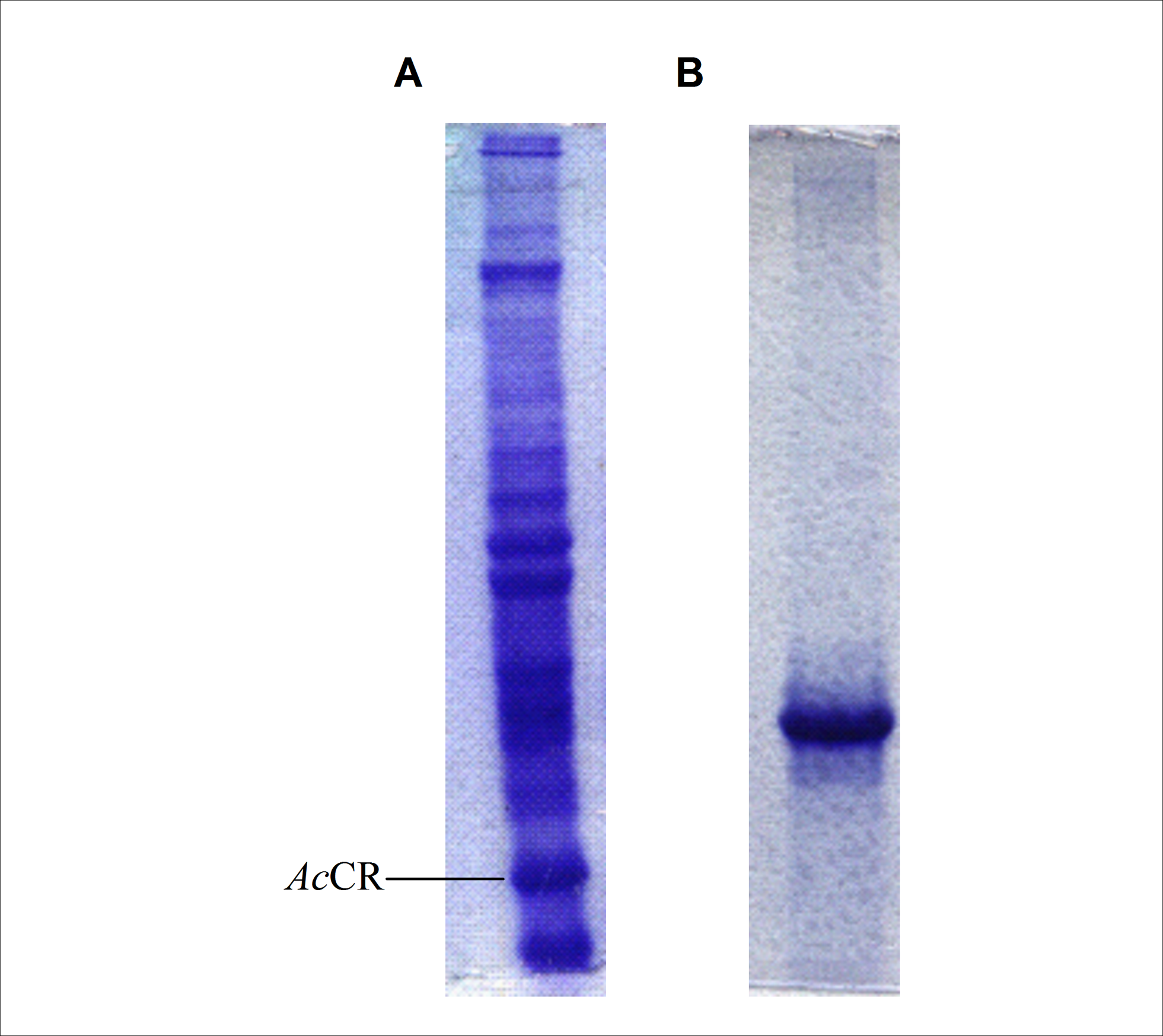

Supplement: Figure S1 — Clear Native-PAGE for purification of Ac CR. (A) Crude enzyme after purification with DEAE-Sepharose; (B) AcCR. The separating and stacking gels contained 9% and 5% acrylamide, respectively. The electrophoresis was performed at 4°C. The target segment (A) was chopped into small pieces, transferred into a dialysis bag with a cutoff molecular weight of 11 kDa and electrophoresed for 10 min to recollect the enzyme. The purified AcCR was subsequently desalted and concentrated by ultrafiltration using Centriprep YM-10, which showed a single band by Native-PAGE (B). Gel was stained with 0.05% Coomassie Blue R-250. (TIF) [file pone.0094543.s001.tif]

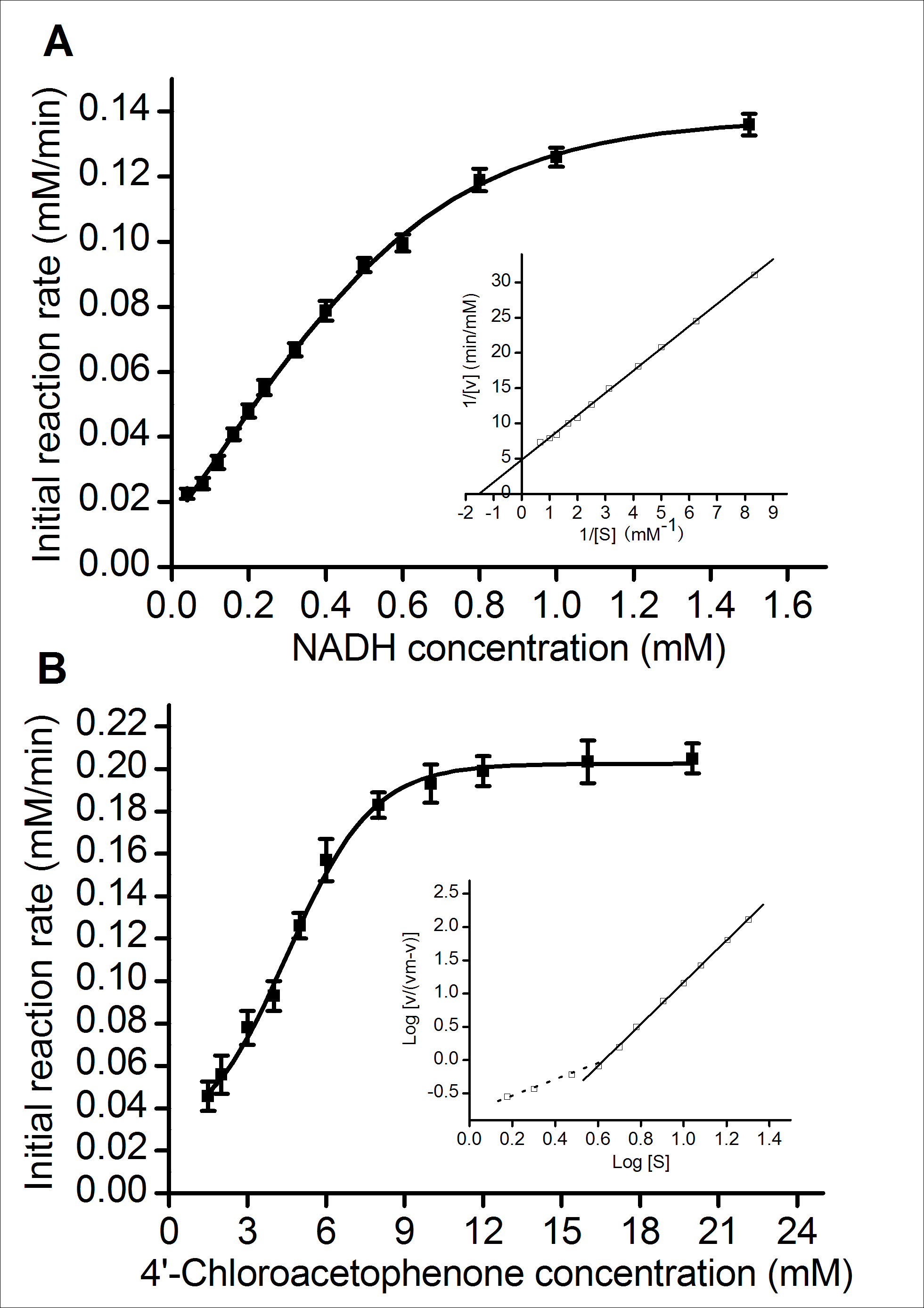

Supplement: Figure S2 — Dependence of Ac CR's activity on concentrations of NADH (A) and 4′-chloroacetophenone (B). Substrate saturation curves were shown with Lineweaver-Burk plot (A) and Hill plot (B) inset. The enzyme's activity was estimated under standard assay conditions, and NADH concentrations varied from 0.04 to 1.50 mM (4′-chloroacetophenone concentration was fixed at 5 mM) and 4′-chloroacetophenone concentrations varied from 1.5 to 20.0 mM (NADH concentration was fixed at 1 mM). The kinetic parameters, Km and Vmax values, were graphically determined from the Lineweaver-Burk and Hill plotting. (TIF) [file pone.0094543.s002.tif]

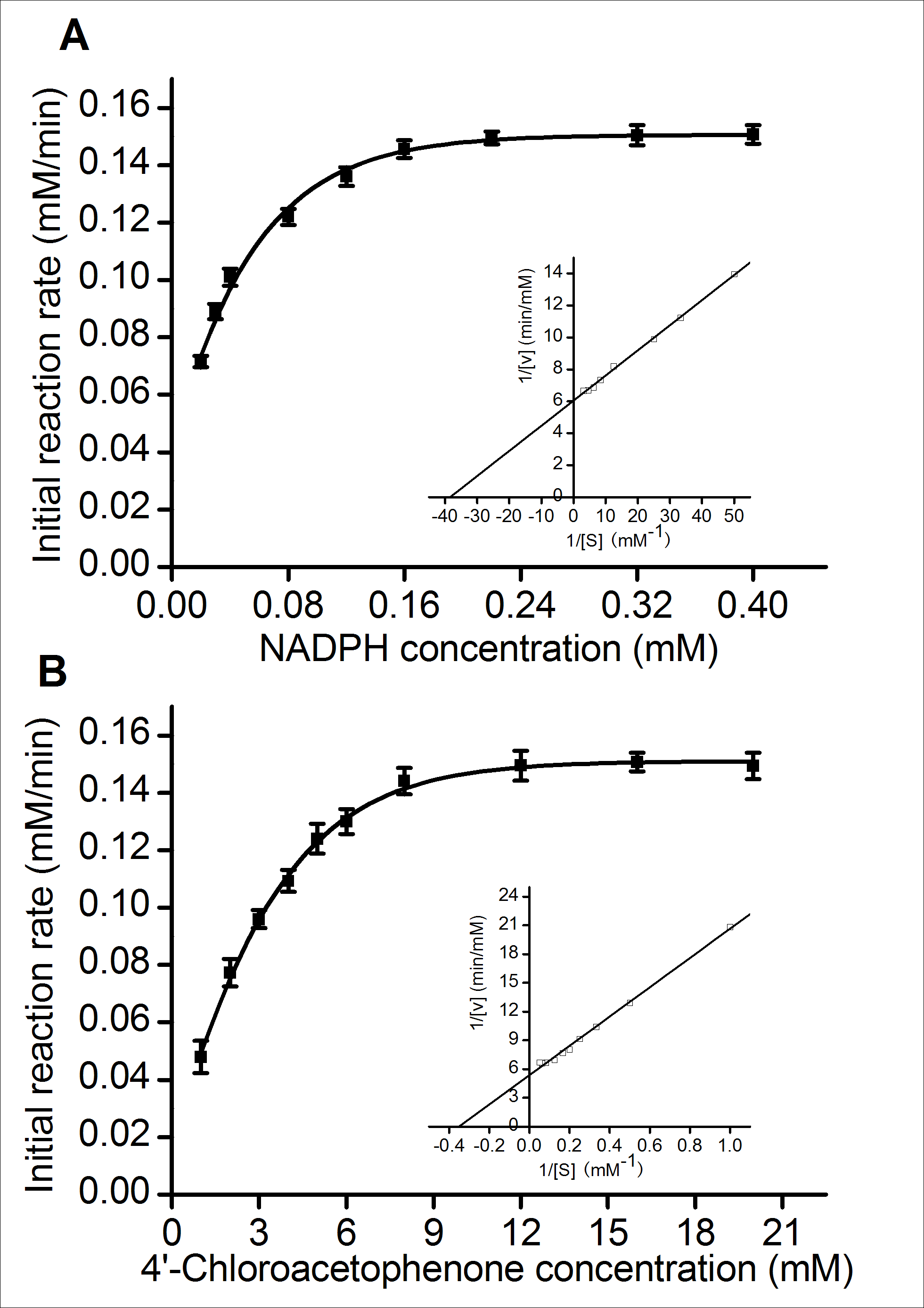

Supplement: Figure S3 — Dependence of Ac CR's activity on concentrations of NADPH (A) and 4′-chloroacetophenone (B). Michaelis–Menten curves were shown with Lineweaver-Burk plots inset. The enzyme's activity was estimated under standard assay conditions, and NADPH concentrations varied from 0.02 to 0.40 mM (4′-chloroacetophenone concentration was fixed at 5 mM) and 4′-chloroacetophenone concentrations varied from 1.5 to 20.0 mM (NADPH concentration was fixed at 0.1 mM). The kinetic parameters, Km and Vmax values, were graphically determined from the Lineweaver-Burk plotting. (TIF) [file pone.0094543.s003.tif]

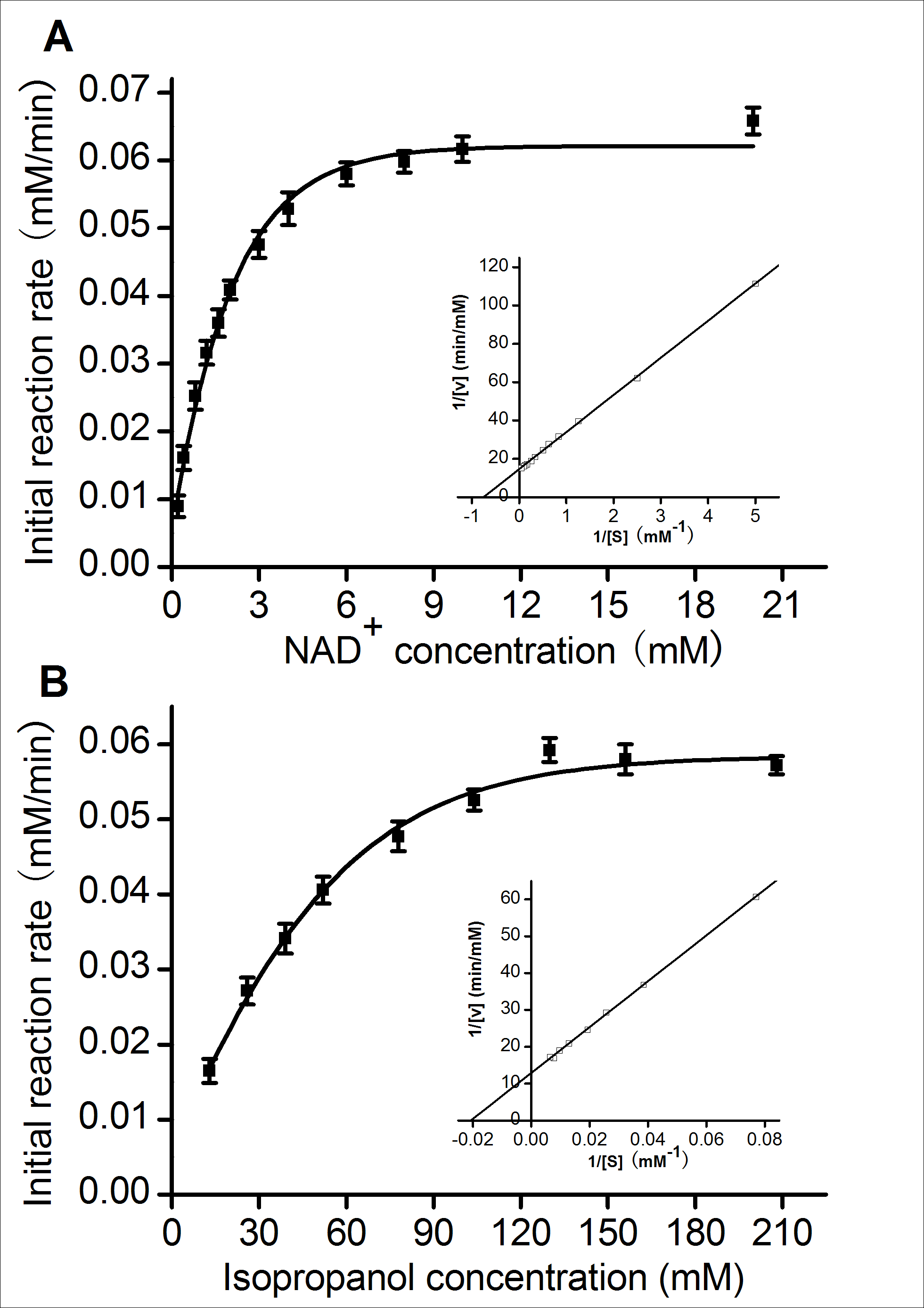

Supplement: Figure S4 — Dependence of Ac CR's activity on concentrations of NAD+ (A) and isopropanol (B). Michaelis–Menten curves were shown with Lineweaver-Burk plots inset. The enzyme's activity was estimated under standard assay conditions, and NAD+ concentrations varied from 0.2 to 20.0 mM (isopropanol concentration was fixed at 110 mM) and isopropanol concentrations varied from 13 to 208 mM (NAD+ concentration was fixed at 5 mM). The kinetic parameters, Km and Vmax values, were graphically determined from the Lineweaver-Burk plotting. (TIF) [file pone.0094543.s004.tif]

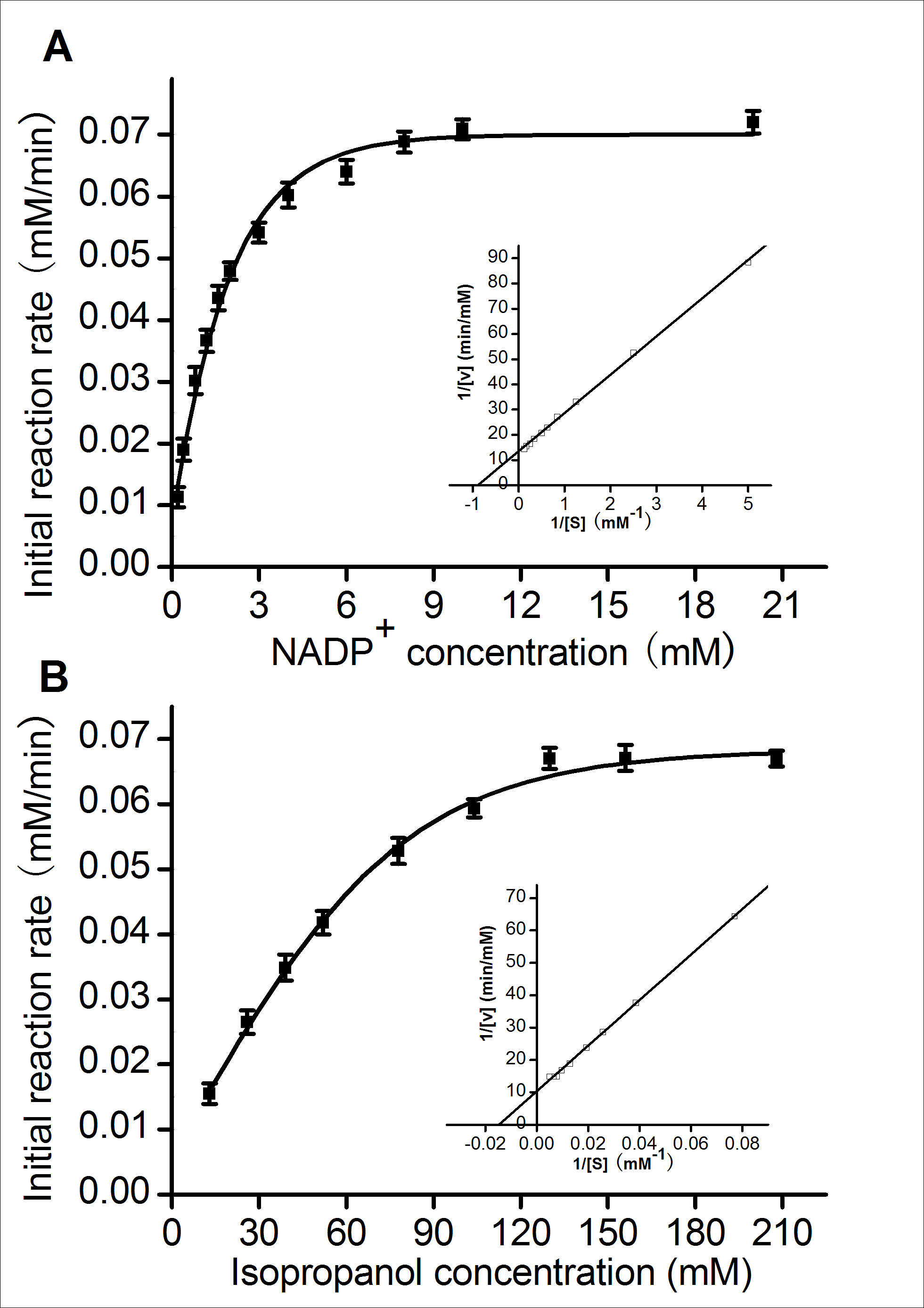

Supplement: Figure S5 — Dependence of Ac CR's activity on concentrations of NADP+ (A) and isopropanol (B). Michaelis–Menten curves were shown with Lineweaver-Burk plots inset. The enzyme's activity was estimated under standard assay conditions, and NADP+ concentrations varied from 0.2 to 20.0 mM (isopropanol concentration was fixed at 110 mM) and isopropanol concentrations varied from 13 to 208 mM (NADP+ concentration was fixed at 5 mM). The kinetic parameters, Km and Vmax values, were graphically determined from the Lineweaver-Burk plotting. (TIF) [file pone.0094543.s005.tif]

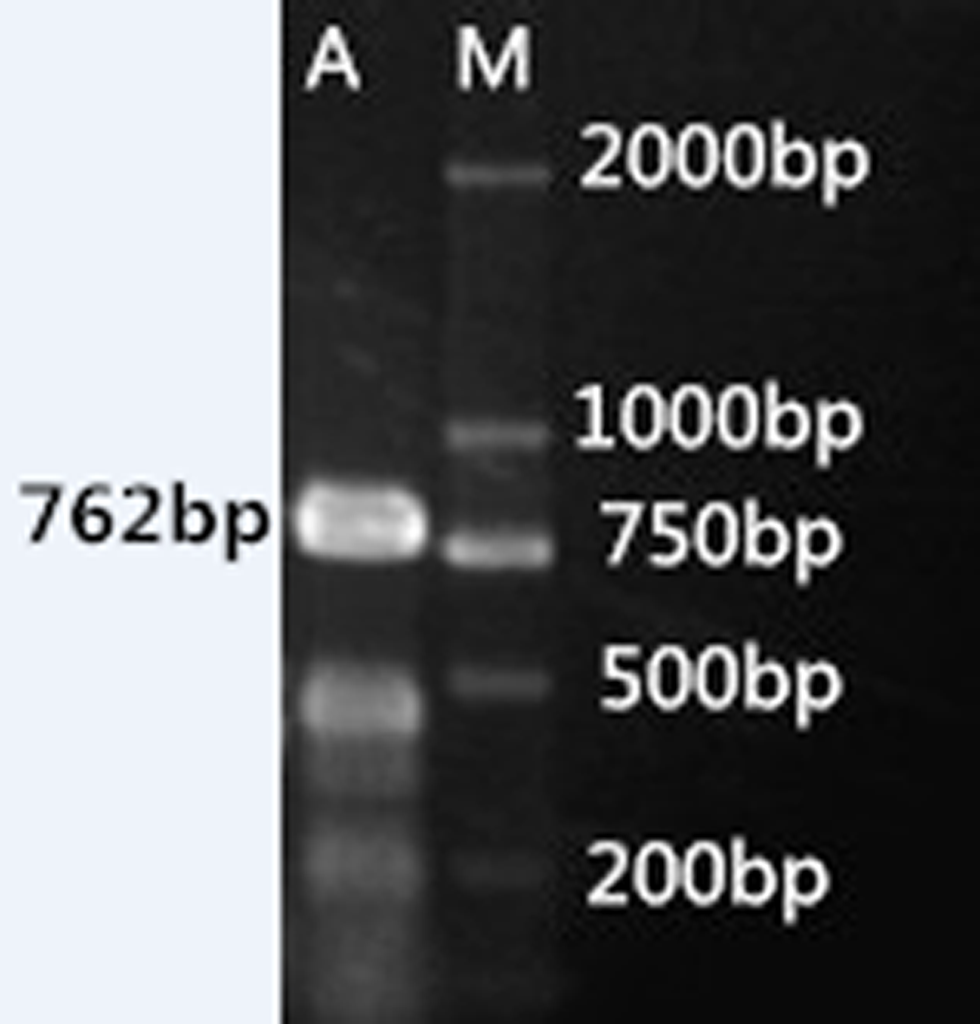

Supplement: Figure S6 — PCR amplification of Ac CR gene sequence. Lane A: 10 µl sample, lane M: marker. (TIF) [file pone.0094543.s006.tif]

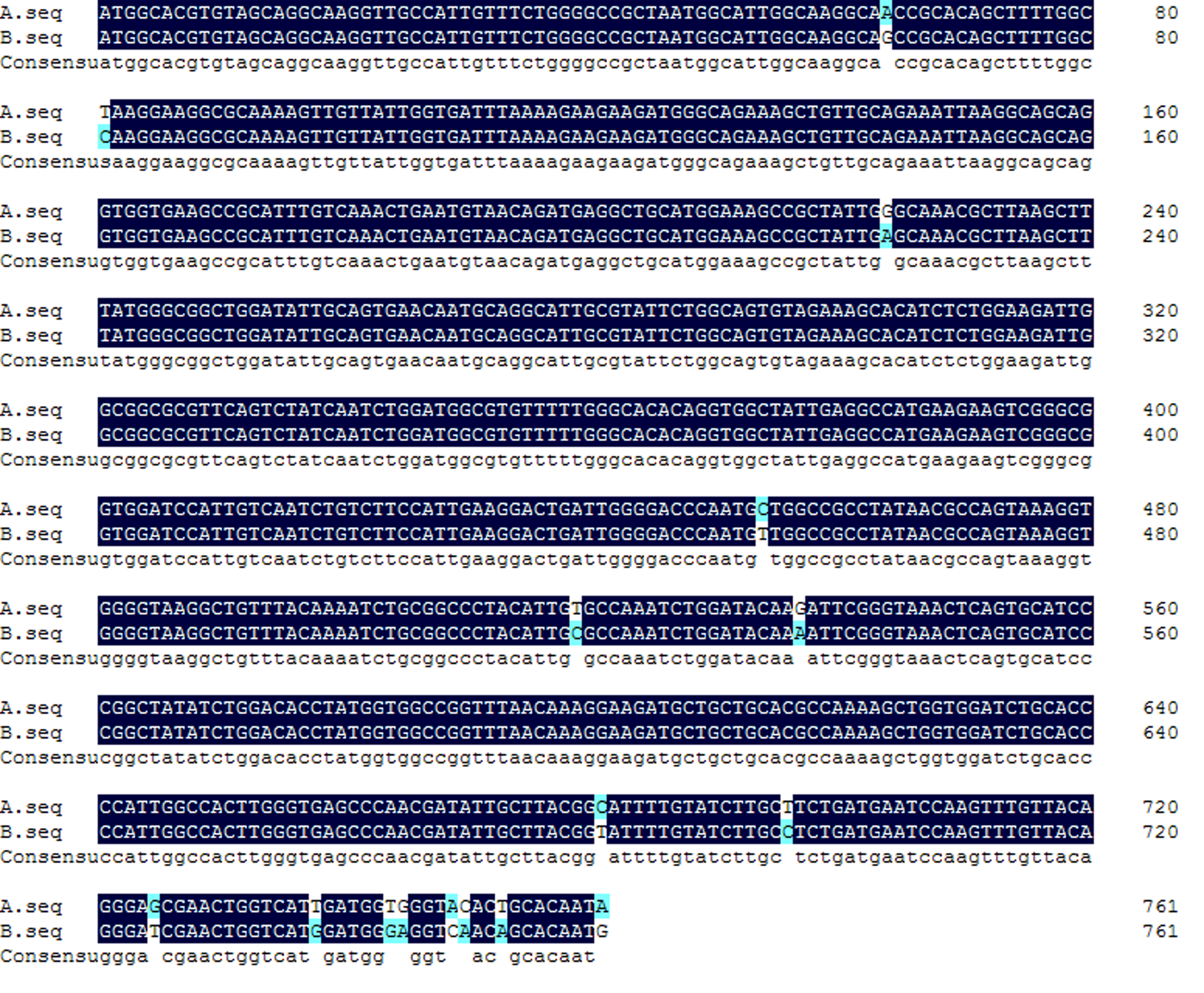

Supplement: Figure S7 — Comparative analysis gene sequences of oxidoreductase from Acetobacter pasteurianus 386B and Ac CR from Acetobacter sp. CCTCC M209061. A: gene sequence of oxidoreductase from Acetobacter pasteurianus 386B; B: gene sequence of AcCR from Acetobacter sp. CCTCC M209061. (TIF) [file pone.0094543.s007.tif]
