# Supplementary material for: A Novel Carbonyl Reductase with Anti-Prelog Stereospecificity from Acetobacter sp. CCTCC M209061: Purification and Characterization
Source: PLoS One. 2014 Apr 16;9(4):e94543. doi: 10.1371/journal.pone.0094543 (PMC3989197; doi:10.1371/journal.pone.0094543)
Supplement: Table S1 — GC analytic methods for various carbonyl compounds and their corresponding chiral alcohols. (DOC) [file pone.0094543.s008.doc]

**Table S1.** GC analytic methods for various carbonyl compounds and their corresponding chiral alcohols.

| Carbonyl compounds | Method | R_t_ for S^a^ (min) | R_t_ for P^b^ (min) |
| --- | --- | --- | --- |
| 2'-Methoxyacetophenone | HP Chiral 20%^d^, 1.0 ml/min, 135°C, 10 min, 1°C/min, 145°C, 2 min | 13.22 | R:15.62; S:16.16 |
| 3'-Methoxyacetophenone | HP Chiral 20%, 1.0 ml/min, 140°C, 10 min, 1°C/min, 145°C, 4 min | 12.40 | R:16.97; S:17.39 |
| 4'-Methoxyacetophenone | HP Chiral 20%, 1.2 ml/min, 140°C, 10 min, 1°C/min, 145°C, 4 min | 16.03 | R:16.42; S:16.74 |
| 4'-Fluoroacetophenone | HP Chiral 20%, 1.2 ml/min, 110°C, 5 min, 1°C/min, 120°C, 5 min | 8.84 | R:14.59; S:15.35 |
| 4'-Chloroacetophenone | HP Chiral 20%, 3.0 ml/min, 140°C, 10 min | 4.78 | R:7.67; S:8.01 |
| 4'-Bromoacetophenone | HP Chiral 20%, 1.0 ml/min, 145°C, 10 min, 1°C/min, 150°C, 7 min | 12.55 | R:18.86; S:19.56 |
| 4'-Nitroacetophenone | HP Chiral 20%, 0.9 ml/min, 175°C, 25 min | 10.74 | R:20.99; S:21.80 |
| 4'-Methylacetophenone | CP-ChiraSil-DEX CB^e^, 0.8 ml/min, 140°C, 10min, | 4.84 | R:6.66; S:7.11 |
| Methyl acetoacetate | HP Chiral 10%^f^, 1.0 ml/min, 70°C, 20 min | 17.63 | R: 16.62  S: 16.62 |
| Methyl acetoacetate^c^ | HP Chiral 10%, 0.4 ml/min, 60°C, 60 min | - | R:55.56; S:56.22 |
| Ethyl acetoacetate | HP Chiral 10%, 0.95 ml/min, 80°C, 15 min | 14.89 | R:13.92;  S: 13.92 |
| Ethyl acetoacetate^c^ | HP Chiral 10%, 0.6 ml/min, 55°C, 60 min | - | R:46.98 S:47.56 |
| 2-Pentanone | CP-ChiraSil-DEX CB, 0.8 ml/min, 100°C, 6 min, | 3.07 | R:5.43  S:5.43 |
| 2-Pentanone^c^ | CP-ChiraSil-DEX CB, 0.4 ml/min, 60°C, 12 min | - | R:10.38  S:10.61 |
| 2-Octonane | CP-ChiraSil-DEX CB, 0.8 mL/min, 120°C, 6 min, | 3.60 | R:4.98  S:4.98 |
| 2-Octonane^c^ | CP-ChiraSil-DEX CB, 0.4 ml/min, 80°C, 25 min, | - | R:19.82  S:20.24 |
| 3,3-Dimethyl-2-butanone | CP-ChiraSil-DEX CB, 0.8 ml/min, 80°C, 12 min | 4.14 | R:9.25;  S:9.56 |
| 1-(Trimethylsilyl)ethanone | CP-ChiraSil-DEX CB, 0.8 ml/min, 85°C, 10 min | 3.65 | R:7.43;  S:7.64 |
| 4-(Trimethylsilyl)-3-butyn-2-one | HP Chiral 20%, 1.2 ml/min, 85°C, 13 min | 5.38 | R:10.64  S:10.64 |
| 4-(Trimethylsilyl)-3-butyn-2-one^c^ | HP Chiral 20%, 1.2 ml/min, 85°C, 15 min | - | R: 13.64  S: 13.35 |

^a^ Retention time for substrate

^b^ Retention time for product

^c^ Derivatization with trifluoroacetic anhydride

# ^d^HP Chiral with 20% permethylated β-cyclodextrin, 30 m × 0.25 mm × 0.25 *μ*m, Agilent J&W Scientific, Folsom, CA, USA

# ^e^CP-ChiraSil-DEX CB, 30 m × 0.25 mm × 0.25 *μ*m, Agilent J&W Scientific, Folsom, CA, USA.

# ^f^HP Chiral with 10% permethylated β-cyclodextrin, 30 m × 0.25 mm × 0.25 *μ*m, Hewlett Packard, USA
